# NFAP2, a novel cysteine-rich anti-yeast protein from *Neosartorya fischeri* NRRL 181: isolation and characterization

**DOI:** 10.1186/s13568-016-0250-8

**Published:** 2016-09-15

**Authors:** Liliána Tóth, Zoltán Kele, Attila Borics, László G. Nagy, Györgyi Váradi, Máté Virágh, Miklós Takó, Csaba Vágvölgyi, László Galgóczy

**Affiliations:** 1 Department of Microbiology, Faculty of Science and Informatics, University of Szeged, Közép fasor 52, Szeged, 6726 Hungary; 2 Department of Medical Chemistry, Faculty of Medicine, University of Szeged, Dóm tér 8, Szeged, 6720 Hungary; 3 Institute of Biochemistry, Biological Research Centre, Hungarian Academy of Sciences, Temesvári krt 62, Szeged, 6726 Hungary; 4 Division of Molecular Biology, Biocenter, Medical University of Innsbruck, Innrain 80-82, Innsbruck, 6020 Austria

**Keywords:** *Neosartorya fischeri*, Protein isolation, Cysteine-rich antifungal protein, Anti-yeast activity, *Candida* spp., Protein structure

## Abstract

The increasing incidence of fungal infections and damages due to drug-resistant fungi urges the development of new antifungal strategies. The cysteine-rich antifungal proteins from filamentous ascomycetes provide a feasible base for protection against molds due to their potent antifungal activity on them. In contrast to this, they show no or weak activity on yeasts, hence their applicability against this group of fungi is questionable. In the present study a 5.6 kDa anti-yeast protein (NFAP2) is isolated, identified and characterized from the ferment broth of *Neosartorya fischeri* NRRL 181. Based on a phylogenetic analysis, NFAP2 and its putative homologs represent a new group of ascomycetous cysteine-rich antifungal proteins. NFAP2 proved to be highly effective against tested yeasts involving clinically relevant *Candida* species. NFAP2 did not cause metabolic inactivity and apoptosis induction, but its plasma membrane disruption ability was observed on *Saccharomyces cerevisiae*. The antifungal activity was maintained after high temperature treatment presumably due to the in silico predicted stable tertiary structure. The disulfide bond-stabilized, heat-resistant folded structure of NFAP2 was experimentally proved. After further investigations of antifungal mechanism, structure and toxicity, NFAP2 could be applicable as a potent antifungal agent against yeasts.

**Electronic supplementary material:**

The online version of this article (doi:10.1186/s13568-016-0250-8) contains supplementary material, which is available to authorized users.

## Introduction

From the second half of the 1990s several extracellular cysteine-rich antifungal proteins have been isolated and characterized from filamentous ascomycetes. The main features of this protein group are a cationic character due to a high amount of arginine and lysine residues, a low molecular mass and the presence of six to eight cysteine residues that form three to four intra-molecular disulfide bonds which provide a high stability against protease degradation, high temperature and broad pH range. They show potent antifungal activity against several opportunistic human, animal, plant and food-borne pathogenic filamentous fungi (Marx 2004; Meyer 2008; Galgóczy et al. 2010; Delgado et al. 2015). Based on in vitro and in vivo interaction and toxicological studies (Marx 2004; Marx et al. 2008; Meyer 2008; Galgóczy et al. 2010; Palicz et al. 2013) the members of this protein group represent exceptionally suitable compounds of commercial drugs, biopesticides and preservatives against molds and offer an alternative, safely applicable solution for recent antifungal challenges in the medicine (Miceli and Lee 2011), agriculture (Magan et al. 2011), biodiversity (Fisher et al. 2012) and cultural heritage protection (Sterflinger and Pinzari 2012).

Beside the strong inhibitory effect on molds, weak anti-yeast activity is described at relative high concentrations of few cysteine-rich antifungal proteins from filamentous ascomycetes, *i.e. Aspergillus niger* antifungal protein (ANAFP; Lee et al. 1999), *Fusarium polyphialidicum* antifungal protein (FPAP; Galgóczy et al. 2013a) belonging to *Penicillium chrysogenum* antifungal protein (PAF)-cluster proteins, and *Penicillium brevicompactum* bubble protein (BP) from the BP-cluster proteins (Seibold et al. 2011).

In our previous work we demonstrated that the filamentous ascomycete *Neosartorya fischeri* (anamorph: *Aspergillus fischerianus*) NRRL 181 isolate secretes a representative of the PAF-cluster proteins. We termed it *Neosartorya fischeri* antifungal protein (NFAP) (Kovács et al. 2011), which effectively inhibits the growth of numerous filamentous ascomycetes (Virágh et al. 2014), but it proved to be ineffective against yeasts.

In the present study a novel cysteine-rich antifungal protein (*Neosartorya fischeri* antifungal protein 2, NFAP2) with high anti-yeast activity was isolated and identified from the supernatant of *N. fischeri* NRRL 181 cultivated in a minimal medium. NFAP2 was characterized concerning its phylogenetic relationship with the other ascomycetous cysteine-rich antifungal proteins, antifungal effect, thermal stability, and structure.

## Materials and methods

### Strains and media

A minimal medium (MM: 2 % sucrose, 0.3 % NaNO_3_, 0.05 % KCl, 0.05 % MgSO_4_ × 7 H_2_O, 0.005 % FeSO_4_ × 7 H_2_O (w/v), 0.25 % 1 M potassium phosphate buffer pH 5.8, 0.01 % trace element solution (v/v); trace element solution: 0.1 % FeSO_4_ × 7 H_2_O, 0.9 % ZnSO_4_ × 7 H_2_O, 0.4 % CuSO_4_ × 5 H_2_O, 0.01 % MnSO_4_ × H_2_O, 0.01 % H_3_BO_3_, 0.01 % Na_2_MoO_4_ × 2 H_2_O (w/v)) was used to produce NFAP2 by *N. fischeri* NRRL 181 strain (Agricultural Research Service Culture Collection, National Center for Agricultural Utilization Research, Peoria, Illinois USA).

The antifungal activity of NFAP2, NFAP, and conventional antifungal agents was investigated against nine yeasts (*Candida albicans* American Type Culture Collection, Manassas, VA, USA, ATCC 10231; *Candida glabrata* Centraalbureau voor Schimmelcultures, Utrecht, The Netherlands, CBS 138; *Candida guilliermondii* CBS 566; *Candida krusei* CBS 573; *Candida lusitaniae* CBS 6936; *Candida parapsilosis* CBS 604; *Candida tropicalis* CBS 94; *Saccharomyces cerevisiae* Szeged Microbiological Collection, Szeged, Hungary, SZMC 0644; and *Schizosaccharomyces pombe* SZMC 0142), and three NFAP-sensitive filamentous fungal isolates (*Aspergillus nidulans* Fungal Genetics Stock Center, Kansas, MO, USA, FGSC A4; *Aspergillus niger* SZMC 601; *Rhizomucor miehei* CBS 360.92) (Kovács et al. 2011; Virágh et al. 2015). Susceptibility tests were performed in low cationic broth medium (LCM: 0.5 % glucose, 0.025 % yeast extract, 0.0125 % peptone (w/v)).

Filamentous fungi were maintained on malt extract agar slants (MEA: 0.5 % malt extract, 0.25 % yeast extract, 1 % glucose, 2 % agar (w/v)), yeasts were maintained on yeast extract glucose medium (YEGK: 1 % glucose; 1 % KH_2_PO_4_; 0.5 % yeast extract, 2 % agar (w/v)) at 4 °C.

### Isolation and purification of NFAP2

NFAP2 was isolated from the supernatant of *N. fischeri* NRRL 181 culture, which was grown in MM. Five 1 l-Erlenmeyer flasks each containing 200 ml MM was inoculated with 2 × 10^7^ conidia and incubated for 7 days at 25 °C under continuous shaking at 210 rpm. Mycelia were removed with filtering the culture through paper filter (Rotilabo-round filters, type 111A; Carl Roth KG, Karlsruhe, Germany), then the mycelia-free supernatant was centrifuged (10,000×*g*, 17 °C) and filtered through paper filter (Fisherbrand QL115 folded filter paper, Fisher Scientific, Pittsburgh, PA, USA) again. NFAP2 was purified from this mycelia-free supernatant based on the slightly modified method described at NFAP previously (Virágh et al. 2014). The <30 kDa molecular fraction of the supernatant was separated by ultrafiltration (Ultracell 30 kDa Ultrafiltartion Discs, regenerated cellulose; Millipore, Billerica, MA, USA) then its protein content was purified by cation-exchange chromatography on a Bio-Scale™ Mini Macro-Prep^®^ High S column (Bio-Rad Laboratories, Hercules, CA, USA) using the BioLogic Duo Flow™ system (Bio-Rad Laboratories, Hercules, CA, USA). The column was equilibrated with 10 mM sodium phosphate buffer (pH 6.6) containing 25 mM NaCl and 0.15 mM EDTA. Bound proteins were eluted with NaCl gradient (0.0–1.5 M) prepared in 10 mM sodium phosphate buffer (pH 6.6) at a flow rate of 1.2 ml min^−1^. The quality of the NFAP2 fractions was checked by SDS-PAGE (Novex™ 18 % Tris–Glycine Mini Protein Gels, 1.0 mm, 10-well; Thermo Fisher Scientific, Waltham, MA, USA). Protein bands were visualized applying Coomassie Brilliant Blue R-250 and silver staining. The pool of the pure NFAP2 fractions was dialyzed (Snake Skin™ dialysis tubing, 3.5 K MWCO, Thermo Scientific, Logan, UT, USA) against double distilled water, then lyophilized and dissolved in double distilled water. This protein solution was sterilized by syringe filtration (Millex-GV, PVDF, pore size: 0.22 µm; Millipore, Billerica, MA, USA).

### Identification of NFAP2

Molar mass measurement of NFAP2 was performed on a Micromass Q-TOF Premier mass spectrometer (Waters MS Technologies, Manchester, UK) equipped with a nanoelectrospray ion source. Partial sequence of NFAP2 was determined from enzymatic digested protein sample. Ten microliter of protein solution containing 1 μg μl^−1^ protein was mixed with a buffer containing 25 mM NH_4_HCO_3_, pH 8.0, reduced with 10 mM DTT and alkylated with 55 mM iodoacetamide. The reduced and alkylated protein was purified with C4 containing ZipTip pipette tip (Millipore, Billerica, MA, USA) and it was subjected to enzymatic cleavage with 0.1 μg trypsin (Promega, Madison, WI, USA) solution (in 25 mM NH_4_HCO_3_) overnight at 37 °C. Then a mass spectrometric (MS) method was used, which was based on the database searching (Mascot Search Engine, NCBInr Database) of the protein fragment from the enzymatic digestion. The digested sample was analyzed on a Waters NanoAcquity UPLC (Waters MS Technologies, Manchester, UK) system coupled with a Micromass Q-TOF premier mass spectrometer. LC conditions were the followings: flow rate: 350 nl min^−1^; eluent A: water with 0.1 % (v/v) formic acid, eluent B: acetonitrile with 0.1 % (v/v) formic acid; gradient: 40 min, 3–40 % (v/v) B eluent; column: Waters BEH130 C18 75 lm 250 mm^−1^ column with 1.7 μm particle size C18 packing (Waters, Milford, MA, USA). The mass spectrometer was operated in MSE and DDA mode with lockmass correction (standard: Glu-1-Fibrinopeptide M + 2H + m/z = 785.842). Acquired data derived from the enzymatic cleavage were processed by the ProteinLynx Global Server (Waters, Milford, MA, USA).

### *In silico* investigations

The SignalP1 4.1 server was used to predict the cleavage site of the signal sequence (Petersen et al. 2011). The molecular weight, pI, grand average of hydropathy (GRAVY) value, total net charge, and disulfide bridge pattern of the mature NFAP2 were predicted by ExPAsy ProtParam tool (Gasteiger et al. 2005), Protein Calculator v3.4 server (The Scripps Research Institute; http://www.scripps.edu/~cdputnam/protcalc.html), and DISULFIND Cysteines Disulfide Bonding State and Connectivity Predictor server (Ceroni et al. 2006), respectively.

### Phylogenetic analysis

The BioEdit program (Hall 1999) was used to examine the antifungal protein sequences. Similarity searches to NFAP2 in the NCBI, EXPASY and JGI databases were performed using the Basic Local Alingment Search Tool (BLAST; Pevsner 2009). All previously described, isolated and characterized cysteine-rich antifungal proteins from filamentous ascomycetes, and the identified putative NFAP2 homologs were involved in the phylogenetic studies. Sequences were aligned by using the PRANK (Löytynoja and Goldman 2008). Ambiguously aligned positions were removed by GBlocks (Talavera and Castresana 2007). A maximum likelihood analysis (ML) was carried out under the WAG model of protein evolution with gamma distributed rate-heterogeneity and 1000 bootstrap replicates. Bootstrap percentages were summarized on the ML tree using the SumTrees script of the Dendropy package (Sukumaran and Holder 2010). Bootstrap proportions >70 % were considered as strong support.

### Antifungal susceptibility tests

The in vitro antifungal effect of NFAP2, NFAP, and conventional antifungal agents (Sigma-Aldrich, St Louis, MO, USA) representing polyenes (amphotericin B, AMB), azoles (fluconazole, FLC and itraconazole, ITC), allylamines (terbinafine, TRB), and echinocandins (caspofungin, CSP) against mid-log phase yeast cells (grown up in LCM at 30 °C under continuous shaking at 210 rpm) and conidia or sporangiospores of 4-days-old filamentous fungi was examined in 96-well microtiter plate bioassays by measuring the optical density of the cultures. All conventional antifungal agents were dissolved in 96 % ethanol to prepare stock solutions (10.24 mg ml^−1^). One hundred microliter of purified NFAP2 (0.195–50 µg ml^−1^ in twofold dilution), or NFAP (25–400 µg ml^−1^ in twofold dilution), or antifungal drug (128–0.25 µg ml^−1^ in twofold dilution) diluted in LCM was mixed with 100 µl of 10^5^ cells or conidia or sporangiospores ml^−1^ suspension prepared also in LCM. The flat-bottom plates were incubated for 0, 24, 48 and 72 h at 30 °C (yeasts), 25 °C (*Aspergillus* spp.), or 37 °C (*R. miehei*) without shaking, and then the absorbance (OD_620_) were measured in well scanning mode after shaking the plates for 5 s with a microtiter plate reader (SPECTROstar Nano, BMG Labtech, Ortenberg, Germany). Fresh medium (200 µl LCM) was used for background calibration. For calculation of the growth ability in the presence of antifungal proteins or drugs, the absorbance of the untreated control cultures (100 µl LCM mixed with 100 µl of 10^5^ cells or conidia or sporangiospores ml^−1^ suspension prepared in LCM) were set to be 100 % growth. The minimal inhibitory concentration (MIC) was defined as the lowest antifungal protein or drug concentration at which growth was not detected after 24 (yeasts and *R. miehei*) or 48 h (*Aspergillus* spp.) of incubation on the basis of the OD_620_ values as compared to the untreated control. All susceptibility tests were repeated three times with three replicates.

### Investigation of the manifestation of antifungal mechanism

All investigations were performed on mid-log phase *S. cerevisiae* cells grown up in LCM at 30 °C under continuous shaking at 210 rpm. To reveal the short- and long-term antifungal effect of NFAP2, 10^5^ cells ml^−1^ were incubated in fresh LCM broth supplemented with the lethal (0.195 µg ml^−1^) or sublethal (0.098 µg ml^−1^) concentration of NFAP2 for 10, 30, 60 min and 4, 6 and 16 h at 30 °C. LCM without NFAP2 was used as control.

To compare the metabolic activity of the NFAP2-treated and untreated cells, FUN1 viability staining (Thermo Fisher Scientific, Waltham, MA, USA) was used based on the manufacturer’s instructions.

To determine the proportion of apoptotic and necrotic cells in NFAP2-treated and untreated samples the Annexin V-FITC (fluorescein isothiocyanate) Apoptosis detection kit (Sigma-Aldrich, St Louis, MO, USA) was used following the manufacturer’s instructions.

Plasma membrane disrupting activity of NFAP2 was investigated by applying the membrane impermeant, red-fluorescent nuclear and chromosome stain propidium iodide (PI). Cells were washed with LCM, and then stained with 5 µg ml^−1^ PI for 10 min at room temperature in the dark, and then washed again with LCM. Cells treated with 70 % (v/v) ethanol for 30 min at 4 °C were used as positive staining control.

Total and Annexin- or PI-positive cell numbers were determined in a Bürker chamber. All experiments were performed in three independent replicates.

### Microscopy

Cells were visualized by light and fluorescence microscopy (Carl Zeiss Axiolab LR 66238C; Zeiss, Oberkochen, Germany) and photographed with a microscope camera (Zeiss AxioCam ERc 5 s; Zeiss, Oberkochen, Germany).

### Heat stability investigation

Heat stability of NFAP2 was investigated on *S. cerevisiae* in microtiter plate bioassay. NFAP2 diluted in LCM (0.78–0.049 µg ml^−1^ in twofold dilution) was continuously heated from 25 to 95 °C, and then was incubated at the final temperature for 5 min. After cooling down to room temperature for 30 min, 100 µl treated protein solution was mixed with 100 µl 10^5^ mid-log phase cells ml^−1^ grown up at 30 °C under continuous shaking at 210 rpm and diluted in LCM, then filled in the well of a flat-bottom microtiter plate. The microtiter plate was incubated at 30 °C for 24 h without shaking. The growth ability of *S. cerevisiae* was determined as described previously in the antifungal susceptibility tests. Untreated NFAP2 and *S. cerevisiae* culture (100 µl LCM mixed with 100 µl 10^5^ ml^−1^ mid-log phase cells) were used as activity and growth controls, respectively. The test was repeated three times with two replicates.

### Electronic circular dichroism spectroscopy and structural investigation

Secondary structure and thermal stability of NFAP2 was examined by electronic circular dichroism (ECD) spectroscopy. Measurements were performed in the 195–260 nm wavelength range using a Jasco-J815 spectropolarimeter (JASCO, Tokyo, Japan). The protein sample was presented in pure water in approximately 0.1 mg ml^−1^ concentration in a 0.1 cm path length quartz cuvette. First, the ECD spectrum of the sample was recorded at 25 °C with a scan speed of 100 nm s^−1^. The temperature was then gradually increased up to 95 °C at a rate of 1 °C min^−1^ using a Peltier thermo electronic controller (TE Technology, Traverse City, MI, USA), while ellipticity data was recorded as a function of temperature at three wavelengths, appointed by the extrema of the spectrum measured at 25 °C. The system was allowed to equilibrate for 1 min, before measurements were taken at each temperature point.

The resultant melting curves were fitted with a symmetrical sigmoidal function of which inflexion point corresponds to the melting temperature (T_m_) of the protein structure.

At the final temperature, 95 °C, ECD spectrum in the 195-260 nm range was recorded again and then the sample was left to cool to 25 °C. Further spectrum acquisitions were done at 25 °C 1 min after cooling, then after 72 h and then 4 weeks later. The reported spectra are accumulations of ten scans, from which the similarly recorded, corresponding solvent spectrum was subtracted. Ellipticity data are given in mdeg units.

For determination of possible disulfide bond pattern of NFAP2, reversed-phase high performance liquid chromatography (RP-HPLC) runs were carried out on a Phenomenex Jupiter C18 column (250 × 4.6 mm; 10 μm particle size; 300 Å pore size; Phenomenex, Torrance, CA, USA) using an Agilent 1100 Series liquid chromatograph (Agilent Technologies, Little Falls, DE, USA). Linear gradient elution was carried out with 0.1 % (v/v) TFA in water (eluent A) and 80 % (v/v) acetonitrile and 0.1 % (v/v) TFA is water (eluent B) from 5 to 40 % (v/v) (B) over 35 min at a flow rate of 1.0 ml min^−1^.

### Statistical analysis

Statistical analysis was performed using Microsoft Excel 2010 software (Microsoft, Edmond, WA, USA). Two sample *t* test was used to reveal significance between treated and untreated samples.

## Results

### Isolation and identification of NFAP2

**Fig. 1:**
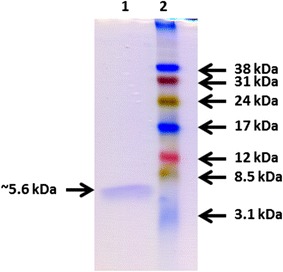
Appearance of the NFAP2 in 18 % (w/v) tris–glycine sodium dodecyl sulfate–polyacrylamide gel (Novex™ 18 % Tris–Glycine Mini Protein Gels, 1.0 mm, 10-well; Thermo Fisher Scientific, Waltham, MA, USA) stained with Coomassie Brilliant Blue R-250. *Lane 1*: purified NFAP2 (1.5 μg), *lane 2*: Low-range Amersham Rainbow Marker (GE Healthcare Life Sciences, Little Chalfont, UK)

**Fig. 2:**
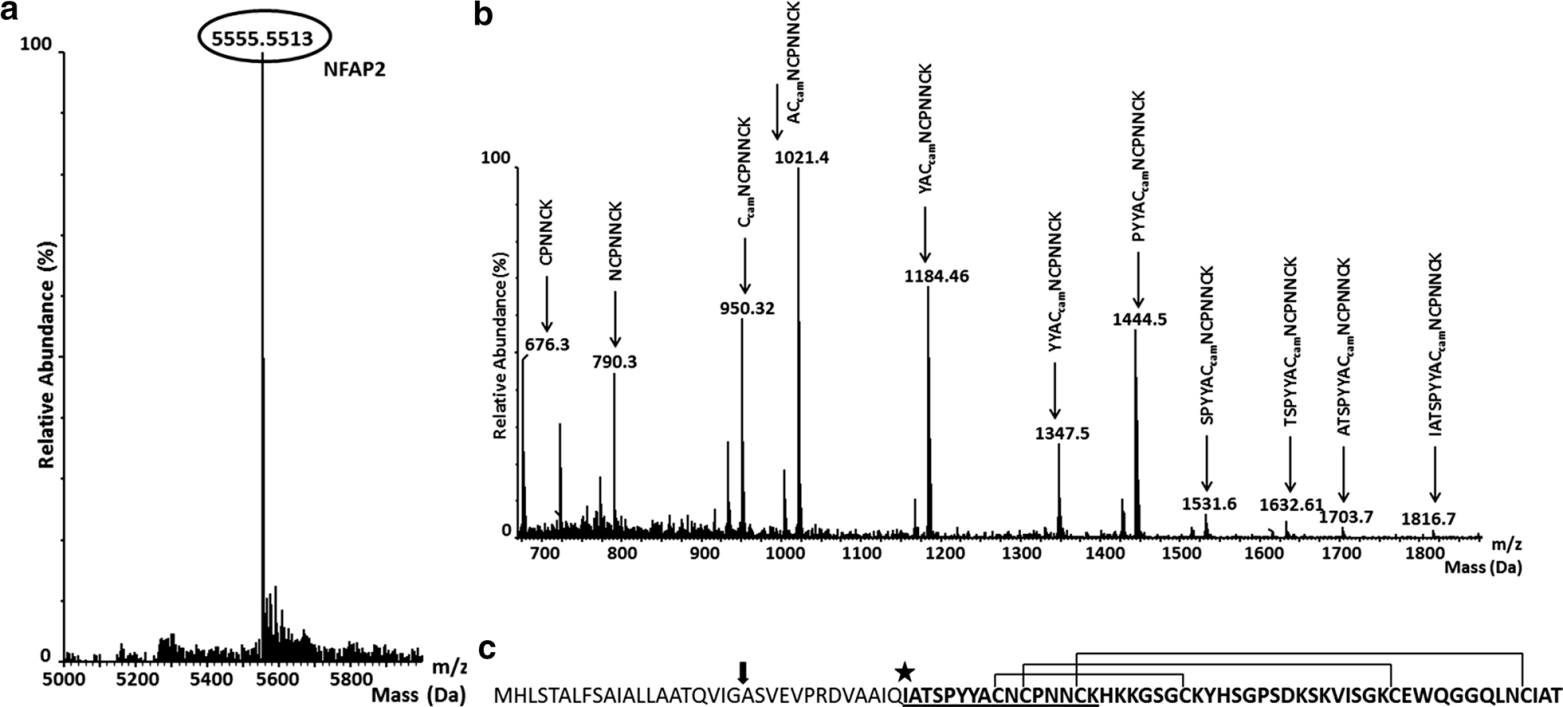
ESI-MS (electrospray ionization mass spectrometry) spectrum of the purified NFAP2 (**a**), and MS/MS spectrum of its N-terminal IATSPYYACNCPNNCK peptide fragment where the first ten amino acids were identified (**b**). In this peptide C11 and C15 formed an internal disulfide bridge due to the incomplete carbamidomethylation. Amino acid sequence of NFAP2 (**c**), where *arrow* indicates the cleavage site of the predicted signal sequence, the first amino acid of the mature protein is marked by an *asterisk*. Identified peptide by the MS analysis of enzyme digested NFAP2 is *underlined*. Connected cysteines indicates the predicted disulfide bridge formations

*N. fischeri* NRRL 181 secreted an anti-yeast protein into the supernatant when it was cultivated in MM. After purification, protein gel electrophoresis revealed the presence of a ~5.6 kDa protein in the pooled fractions which showed anti-yeast activity (Fig. 1). Presence of any other proteins (even NFAP) was not detected with silver staining (Additional file 1: Fig. S1) and MS analysis in the purified sample. Mass spectrometric molar mass measurement of this protein resulted 5555.5513 Da (Fig. 2a) which showed good correlation with its appearance on the protein gel (Fig. 1). The average yield of purified NFAP2 was 368 ± 19 µg l^−1^ (n = 5).

Processed data from MS analysis of enzymatic digested NFAP2 was subjected to database searching using Mascot search engine. This revealed a partial sequence of the examined protein. An IATSPYYACNCPNNCK peptide fragment of a hypothetical protein from *N. fischeri* NRRL 181 was identified (Fig. 2b).

BLAST search for this identified fragment on the submitted *N. fischeri* NRRL 181 genome in the UniProt and NCBI databases resulted in an uncharacterized, hypothetical protein (accession numbers: A1DBL3 and XP_001262150.1, respectively) (Fig. 2c). The measured molecular mass of the purified anti-yeast protein corresponded well to the calculated mass of the IATSPYYACNCPNNCKHKKGSGCKYHSGPSDKSKVISGKCEWQGGQLNCIAT fragment of this hypothetical protein. The identified 5.6 kDa-protein was termed as *Neosartorya fischeri* antifungal protein 2 (NFAP2), and its encoding cDNA sequence was submitted in the EMBL-EBI ENA nucleotide sequence database under LT160067 accession number.

### In silico investigations of NFAP2

Based on in silico investigations NFAP2 is expressed as an 86 amino acid length pre-pro protein and 21 amino acids length extracellular signal sequence, and additional 13 amino acids are cleaved from the N-terminal end of the protein during the maturation process (Fig. 2c). The mature form of NFAP2 consists of 52 amino acids (Fig. 2c) and has a calculated molecular mass of 5564.3 Da and pI of 9.02. NFAP2 is hydrophilic (GRAVY = −0.731) and positively charged (net charge at pH 7.0 = + 5.2) in the consequence of the R/K/D/E = 0/7/1/1 amino acid ratio. The six cysteines at position of 9, 11, 15, 23, 40 and 49 form three disulfide bridges between C9 and C23, C11 and C40, C15 and C49 showing *abcabc* pattern.

### Phylogenetic relationships of NFAP2 to other antifungal proteins

**Fig. 3:**
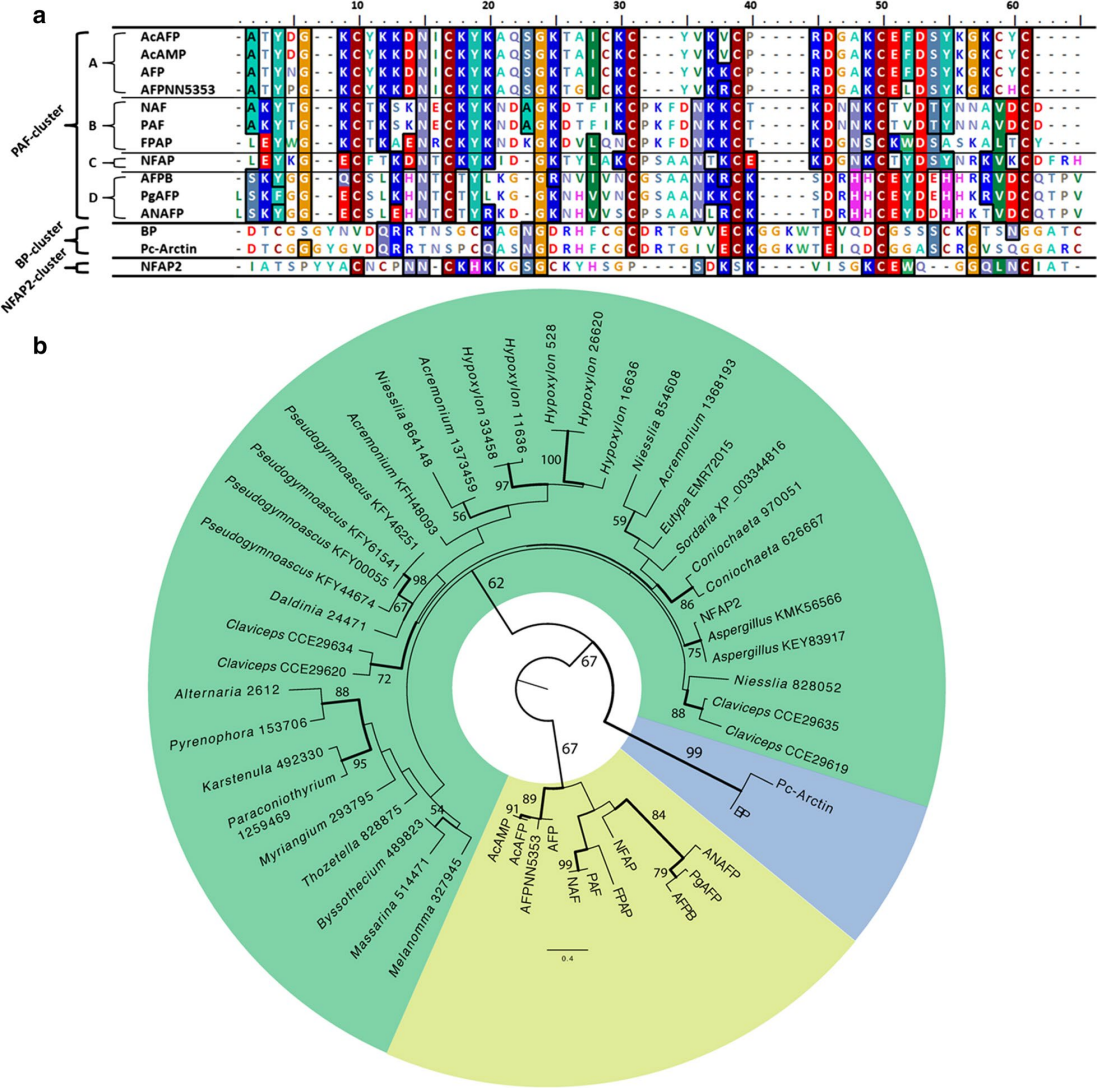
Alignment of isolated and characterized cysteine-rich antifungal proteins from filamentous ascomycetes (**a**). Based on the amino acid similarity they are separated into three different groups (PAF-, BP-, and NFAP2-cluster proteins), and further four different subgroups are distinguishable in the PAF-cluster proteins. Maximum likelihood tree of isolated PAF- and BP-cluster proteins, and NFAP2 and its homologs detected through BLAST searches in published fungal genomes (**b**). ML *bootstrap* values >50 % are shown next to branches, all *bootstrap* values are available on Additional file 1: Fig. S3. The antifungal proteins included in the phylogenetic analyses separated into three major clades, Group 1 (BP-cluster proteins, *blue*), Group 2 (PAF-cluster proteins, *yellow*) and Group 3 (NFAP2 and its homologs, *green*). The presence of four different subgroups of PAF-cluster proteins is also supported by the phylogenetic tree. Abbreviations of isolated and characterized proteins: AcAFP, *Aspergillus clavatus* VR1 antifungal protein (Acc. No.: A1CSS4), AcAMP, *Aspergillus clavatus* ES1 antimicrobial peptide (Acc. No.: D3Y2M3), AFP, *Aspergillus giganteus* MDH 18894 antifungal protein (Acc. No.: P17737), AFP_NN5353_, *Aspergillus giganteus* A3274 antifungal protein (Acc. No.: not available, Binder et al. 2011); AFPB, *Penicillium digitatum* CECT 20796 antifungal protein (Acc. No.: K9FGI7); ANAFP, *Aspergillus niger* KCTC 2025 antifungal protein (Acc. No.: A2QM98); BP, *Penicillium brevicompactum* Dierckx ‘bubble protein’ (Acc. No.: G5DC88); FPAP, *Fusarium polyphialidicum* SZMC 11042 antifungal protein (Acc. No.: E1UGX4); NAF, *Penicillium nalgiovense* BFE 66, 67, 474 antifungal protein (Acc. No.: not available, Geisen 2000); NFAP, *Neosartorya fischeri* NRRL 181 antifungal protein (Acc. No.: D4YWE1); NFAP2, *Neosartorya fischeri* NRRL 181 antifungal protein 2 (Acc. No.: A1DBL3); PAF, *Penicillium chrysogenum* Q176 antifungal protein (Acc. No.: B6HWK0); Pc-Arctin, *Penicillium chrysogenum* A096 ‘bubble protein’ (Acc. No.: CAP96194); PgAFP, *Penicillium chrysogenum* RP42C antifungal protein (Acc. No.: D0EXD3). The Acc. No. or Protein ID of the putative protein is indicated in the panel **b** at NFAP2 homologs. For further information (species name, sequence, etc.) see Additional file 1: Table S1

Amino acid sequence of the mature NFAP2 shows 11–21 % identity to the described, isolated and characterized PAF-, and BP-cluster cysteine-rich antifungal proteins from filamentous ascomycetes (Fig. 3a). BLAST searches yielded 35 protein sequences with significant similarity to NAFP2 in published *Ascomycota* genomes (Additional file 1: Table S1; Fig. S2). The predicted mature forms of these putative proteins show 35-98 % amino acid identity to NFAP2 (Additional file 1: Fig. S2). Until now, none of these NFAP2 homologs has been isolated and described, NFAP2 is the first one. Next, we were interested in the phylogenetic relationships of NFAP2 to PAF-, and BP-cluster proteins. Thus we inferred the phylogeny of antifungal proteins comprising the NFAP2, its detected homologs, and isolated PAF- and BP-cluster proteins. We found that the antifungal proteins included in the analyses separated into three major groups, one including the BP-cluster proteins (Group 1—blue in Fig. 3b), a second including PAF-cluster proteins (Group 2—yellow in Fig. 3b) and another that included NFAP2 and its detected homologs (Group 3—green in Fig. 3b). Statistical support for these three clades is moderate, which is not surprising given the short alignable sequences of these proteins. However, the inferred grouping was consistent across several analyses and with previous phylogenetic results (Seibold et al. 2011; Galgóczy et al. 2013a; Garrigues et al. 2016). It is noteworthy that Garrigues et al. (2016) distinguished two groups in the clade containing PAF-related proteins (Group 2—yellow in Fig. 3b). These two groups are discernable on our phylogeny too, although they do not form monophyletic sister clades. Taken together, observations made by Garrigues et al. (2016) and by us are consistent with the existence of four groups, although rigorous testing of the existence of class-level clades will require closer scrutiny in future studies. Nevertheless, our results clearly indicate that NFAP2 forms a new, phylogenetically distinct group among antifungal proteins found in *Acremonium*, *Alternaria*, *Aspergillus*, *Byssothecium*, *Claviceps*, *Coniochaeta*, *Daldinia*, *Eutypa*, *Fusarium*, *Hypoxylon*, *Karstenula*, *Massarina*, *Melanomma*, *Myriangium*, *Neosartorya*, *Niesslia*, *Penicillium*, *Paraconiothyrium*, *Pseudogymnoascus*, *Sordaria*, and *Thozetella* species suggesting that NFAP2 homologs function in a phylogenetically diverse set of *Ascomycota* fungi.

### Antifungal susceptibility tests

MICs of NFAP2, NFAP and conventional antifungal agents for the investigated fungal isolates are shown in Table 1.

**Table 1:** In vitro minimal inhibitory concentration (MIC) values of NFAP2, NFAP and conventional antifungal agents for the investigated fungal isolates

Fungus	MIC (µg ml^−1^)						
	NFAP2	NFAP	AMB	ITC	FLC	TRB	CSP
Yeasts							
*Candida albicans* ATCC 10231	0.781	>200	8	>64	>64	64	16
*Candida glabrata* CBS 138	0.391	>200	8	>64	>64	64	16
*Candida guilliermondii* CBS 566	0.391	>200	4	2	8	16	16
*Candida krusei* CBS 573	1.563	>200	8	2	>64	64	16
*Candida lusitaniae* CBS 6936	0.781	>200	8	16	>64	16	16
*Candida parapsilosis* CBS 604	0.391	>200	8	2	>64	16	32
*Candida tropicalis* CBS 94	0.391	>200	8	>64	>64	>64	8
*Saccharomyces cerevisiae* SZMC 0644	0.195	>200	4	2	64	>64	16
*Schizosaccharomyces pombe* SZMC 0142	0.391	>200	4	8	64	>64	16
Filamentous fungi							
*Aspergillus nidulans* FGSC A4	>25	200	>64	4	>64	8	>64
*Aspergillus niger* SZMC 601	>25	50	8	8	>64	16	>64
*Rhizomucor mieheii* CBS 360.92	>25	>200	2	1	16	8	8

MIC was determined after 24 h (yeasts and *R. miehei* CBS 360.92) and 48 h (*Aspergillus* spp.) of incubation. Abbreviations: *AMB* amphotericin B, *CSP* caspofungin, *FLC* fluconazole, *ITC* itraconazole, *NFAP Neosartorya fischeri* antifungal protein, *NFAP2 Neosartorya fischeri* antifungal protein 2, *TRB* terbinafine

The MICs of NFAP2 for yeasts were in the range of 0.195–1.563 µg ml^−1^, where *S. cerevisiae* proved to be the most (MIC: 0.195 µg ml^−1^), and *C. krusei* the least (MIC: 1.563 µg ml^−1^) susceptible. MIC values varied between 0.391 and 1.563 µg ml^−1^ for clinically relevant *Candida* species, and these values did not change after prolonged incubation time (at 48 and 72 h). Yeasts were not susceptible to NFAP in its investigated concentration range. In contrast to these results, the applied concentrations of NFAP could inhibit the growth of *A. nidulans* and *A. niger* (MIC: 200 and 50 µg ml^−1^, respectively), and NFAP2 was ineffective against all filamentous fungal isolates in the investigated concentration range. Total growth inhibition of *R. mieheii* was not observed in the presence of NFAP. Previously this strain proved to be slightly sensitive (Kovács et al. 2011) or resistant (Virágh et al. 2014) to this protein depending on the applied test medium.

To compare the efficacy of NFAP2 with the different types of conventional antifungal agents, the susceptibility of yeast isolates were also tested to AMB, CSP, FLC, ITC, and TRB. In the applied concentration range AMB (MIC: 4–8 µg ml^−1^) and CSP (MIC: 8–32 µg ml^−1^) were able to inhibit their growth, but resistances to FLC (MIC: 8– >64 µg ml^−1^), ITC (MIC: 2– >64 µg ml^−1^), and TRB (MIC: 16– >64 µg ml^−1^) were observed, except of *C. guilliermondii* which proved to be sensitive to all antifungal drugs.

### Characterization of the antifungal mechanism of NFAP2

The manifestation of antifungal mechanism of NFAP2 on yeast cells was investigated at its sublethal and lethal concentrations. Physiological changes in cells in the presence of an antifungal can be investigated at its sublethal concentrations which do not kill the fungus.

The two-colour fluorescent FUN1 stains the cytoplasm and metabolically inactive vacuoles green, while the metabolically active ones red. Based on the proportion of the red and green vacuoles between the treated and untreated samples (data not shown), change in the metabolic activity of *S. cerevisiae* was not detected in the presence of sublethal NFAP2 concentration even after 16 h-long NFAP2 treatment.

Annexin V-FITC Apoptosis Detection Kit dyes the apoptotic cells green, while the necrotic cells are counterstained red by the membrane impermeant, red-fluorescent nuclear and chromosome stain PI, and living cells do not show any fluorescence. There was no significant difference between the proportion of green cells in the NFAP2-treated and untreated samples (ca. 1 % of the total cell number) even after 16 h (data not shown). In short exposure time (10, 30 and 60 min) at sublethal NFAP2 concentration, same percent of the cells was counterstained with PI in the treated and untreated samples (ca. 1 % of the total cell number), but after 16 h of incubation three times more red cells were counted in the treated sample than in the untreated control reaching a statistically significant difference (p = 0.00004). Based on this last observation we suggested that NFAP2 cannot induce apoptosis in the yeast cells, but can disrupt the plasma membrane. It was verified with a simple PI-staining.

**Fig. 4:**
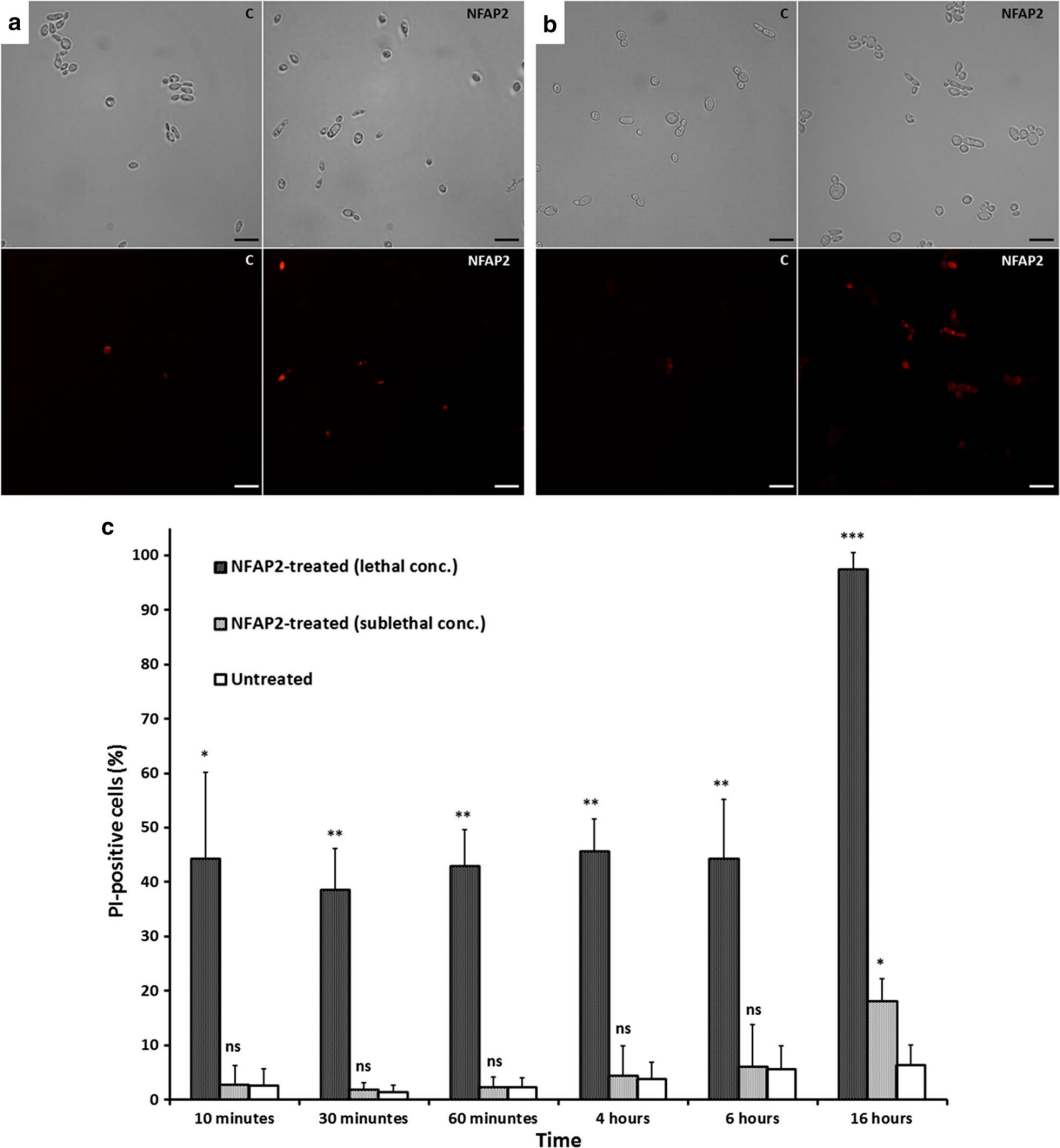
Propidium iodide (PI) staining of *S. cerevisiae* cells after sublethal (0.089 µg ml^−1^) NFAP2 treatment for 16 h at 30 °C (**a**) and lethal (0.195 µg ml^−1^) NFAP2 treatment for 10 min at 30 °C (**b**). Intracellular *red fluorescence* indicates membrane disruption. **c** Untreated control, NFAP2: NFAP2-treated cells. *Scale bars* represent 20 μm. Proportion of the PI-positive *S. cerevisiae* cells in the sublethal and lethal NFAP2-treated and untreated samples after different incubation times at 30 °C (**c**). Significant differences (p-values) were determined based on the comparison with the untreated control. ***p < 0.0001, **p < 0.005, *p < 0.05, *ns* no significant difference

During 6 h exposure to sublethal NFAP2 concentration, same percent of the total cell number showed red fluorescence as in the untreated control. In contrast to this, at 16 h the 6 % of the total cell number were PI-positive in the untreated control compared to the sample treated with sublethal concentration of NFAP2 where it was 18 % (Fig. 4a, c). At this time point the total cell number reduced to ca. 60 percent of the untreated control in the presence of NFAP2 (data not shown). When the cells were exposed to lethal concentration of NFAP2, significant differences were observed in the number of PI-positive cells between the NFAP2-treated sample and the untreated control already after 10 min of incubation (Fig. 4b, c). After 16 h, viable cells were not observed in the NFAP2-treated sample (Fig. 4c).

Taking these observations into consideration the antifungal mechanism of NFAP2 results in plasma membrane disruption and the timing of this activity depends on the applied concentration.

### Thermal stability of NFAP2 and preliminary structural investigations

After continuous heating and 5 min exposure at 95 °C, NFAP2 maintained its antifungal activity against *S. cerevisiae* with a one dilution step shift in the MIC from 0.195 to 0.391 µg ml^−1^ presumably due to its folded and disulfide bond-stabilized compact tertiary structure. To prove this hypothesis we performed thermal unfolding experiments monitored by far-UV electronic circular dichroism (ECD) spectroscopic measurements and a conformational RP-HPLC analysis.

**Fig. 5:**
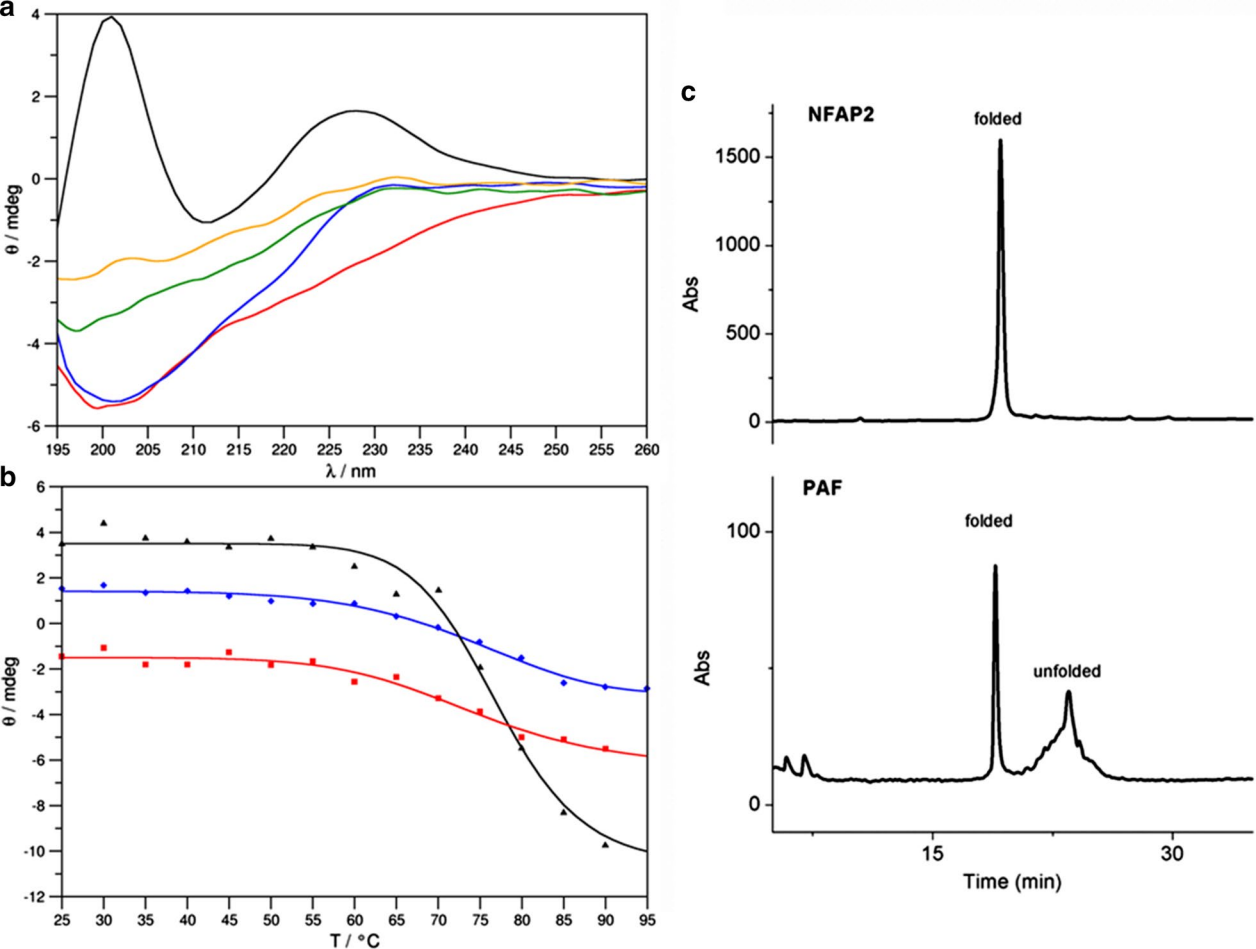
ECD spectra of NFAP2 recorded at 25 °C (*black*), 95 °C (*red*), and again at 25 °C immediately (*blue*), 72 h (*green*) and 4 weeks (*orange*) after heat treatment (**a**). Thermal unfolding curves of NFAP measured at 200 nm (*black*, R^2^ = 0.9899, T_m_ = 76.60 °C), 212 nm (red, R^2^ = 0.9770, T_m_ = 72.49 °C) and 228 nm (*blue*, R^2^ = 0.9910, T_m_ = 74.00 °C) (**b**). RP-HPLC chromatogram of NFAP2 and its comparison with folded and unfolded PAF (**c**)

ECD spectrum of NFAP2 at 25 °C shows features similar to spectra of the homologous PAF protein (Fizil et al. 2015) and other disulfide bridged, β-structured proteins (Lees et al. 2006) (Fig. 5a) The spectrum has two maxima at 200 nm and 228 nm and a low intensity minimum centering at 212 nm. The maximum at 200 nm reflects β-conformation and contributions from the spectral transitions of disulfide bridges. The maximum at 228 nm is mainly attributed to the disulfide bridges while the low intensity minimum at 212 nm is, again, indicates β-conformation. The spectrum measured at 95 °C reflects the total loss of ordered secondary structure. The disappearance of the intense positive band at 228 nm indicates either conformational change (Hider et al. 1988) or the UV light-induced disruption of disulfide bridges at high temperature. It has been reported that UV excitation of aromatic residues may result in electron or H ejection which can reduce adjacent disulfides (Neves-Petersen et al. 2002). After the cooling of NFAP2 solution back to 25 °C moderate structural reorganization takes place, but this reorganization is incomplete even 4 weeks after the annealing. Thermal unfolding curves (Fig. 5b) indicate remarkable thermal stability of this protein. The native fold remains intact up to 70 °C, although thermal denaturation is irreversible.

MS data already indicated that the cysteine residues were oxidized and intra-molecular disulfide bonds could be formed between them. To prove it and reveal the possible disulfide bond pattern of NFAP2 an RP-HPLC method was applied. It is known that RP-HPLC has the ability to correlate the retention behavior of peptides and proteins with their conformation. Based on this finding as well as on our experience on folding of *P. chrysogenum* antifungal protein PAF (Váradi et al. 2013), disulfide bond formation of NFAP2 was followed by reversed-phase liquid chromatography. Figure 5c shows RP-HPLC elution profiles of folded NFAP2 and PAF measured under the same conditions. NFAP2 and PAF share structural similarities concerning their cationic character and the number of amino acids they are composed of. Both proteins are stabilized by three disulfide bridges. The common *abcabc* disulfide bond pattern has already been proven for PAF, and naturally folded PAF was shown to have much smaller retention time on RP-HPLC than any other variant possessing unnatural disulfide bond pattern (Batta et al. 2009; Váradi et al. 2013). Considering that NFAP2 elutes from the reversed-phase column as early as naturally folded PAF, the same interlocking disulfide bond pattern (*abcabc*) seems to be the most probable for NFAP2 as we predicted in silico (Fig. 2c).

## Discussion

In this work we proved that the *N. fischeri* NRRL 181 is able to secrete an anti-yeast protein (NFAP2) in addition to the previously well-characterized, anti-mold NFAP (Kovács et al. 2011; Galgóczy et al. 2013b; Virágh et al. 2014, 2015). When *N. fischeri* NRLL 181 was cultivated in a minimal medium, NFAP2 could be purified from the supernatant with the same single-step method that we applied at NFAP (Virágh et al. 2014), and the presence of NFAP was not observable. Similarly, when *N. fischeri* NRLL 181 was cultivated in a complete medium (Kovács et al. 2011) only the NFAP was present in the supernatant. These results indicate that the antifungal protein profile of *N. fischeri* NRRL 181 highly depends on the cultivation medium.

NFAP2 and its detected putative homologs represent a phylogenetically distinct, new group of cysteine-rich antifungal proteins from filamentous ascomycetes beside the BP- and PAF-cluster proteins (Fig. 3b) and a putative fourth group (see Garrigues et al. 2016), and they are widespread among filamentous ascomycetes (Additional file 1: Table S1; Fig. S2). The antifungal spectrum of PAF- and BP-cluster proteins is differing (Marx 2004; Galgóczy et al. 2010; Seibold et al. 2011; Chen et al. 2013). They have a potent antifungal activity on molds, and show no (Marx 2004; Galgóczy et al. 2010) or weak activity against yeasts (Lee et al. 1999; Seibold et al. 2011; Galgóczy et al. 2013a). In the genome of *N. fischeri* NRRL 181, a putative BP-cluster protein encoding gene was identified, the *Neosartorya fischeri* ‘bubble protein’ (NFBP, Acc. No.: A1DKX1) (Fig. 3a) (Seibold et al. 2011). Based on our in silico a prediction by the SignalP1 4.1 server (Petersen et al. 2011), NFBP is also a secreted protein (data not shown). Considering this arsenal of various extracellular antifungal proteins with different antifungal spectrum, presence of a complex antifungal mechanism in *N. fischeri* is hypothesized to fight against other fungi. The role and other biological function in addition antifungal toxicity is supposed at PAF. It can modulate the asexual development in *P. chrysogenum* (Hegedüs et al. 2011). Similar or other physiological role of NFAP2 is also possible on the native producer, but its demonstration is waiting for further investigations.

NFAP2 was secreted in small yield by *N. fischeri* NRRL 181 into the culture medium comparing to NFAP where three-fold higher yield has been described (Kovács et al. 2011; Virágh et al. 2014). Although, considering that NFAP2 was able to inhibit the growth of different yeasts at its relative low concentrations, this yield is quite high. Different clinically relevant *Candida* species proved to be susceptible to NFAP2, which exerted fungicide activity on them (data not shown). In susceptibility tests clinical reference strain of *C. albicans* and other emerged non-*albicans Candia* (NAC) which can cause serious cutaneous, mucosal and/or systemic infections were involved. At these species moderate or strong primary and secondary resistance to the conventional antifungal drugs is described (Papon et al. 2013). In our susceptibility tests some *Candida* isolates proved to be resistant to one or more conventional antifungal agents. If they were sensitive to them, the antifungal drugs showed higher MIC than NFAP2. The observed extremely low MICs and the fact that none of the investigated yeasts were resistant to NFAP2 render commercial utility as potential antifungal for this protein.

In comparison, the anti-mold NFAP caused reduced metabolic activity and apoptosis induction in the sensitive *Aspergillus nidulans* within a short exposure time (Virágh et al. 2015), while NFAP2 could not that suggests a different mode of action. Based on our observations it could be supposed that the main antifungal mechanism of NFAP2 on yeast cell is the disruption of the plasma membrane, however further experiments are required to prove this and to reveal the exact mode of action. Membrane disruption activity of several cationic antimicrobial peptides from plants and human on *C. albicans* and NAC has been described (Nawrot et al. 2013; Swidergall and Ernst 2014). It is described that the membrane disruption ability of antimicrobial proteins is in tight connection with the high abundance of arginine and lysine residues which render high positive charge to them (Lee and Lee 2015; Tam et al. 2015). Hence the same activity of NFAP 2 is corroborated by its cationic character (net charge is +5.2 at pH 7.0) due to the presence of seven lysine residues.

Similarly to NFAP (Kovács et al. 2011), NFAP2 also proved to be heat-stable owing to its folded and disulfide bridge-stabilized tertiary structure. This structure is common within the group of ascomycetous cysteine-rich antifungal proteins and experimentally proved at AFP (Campos-Olivas et al. 1995; Lacadena et al. 1995); PAF (Batta et al. 2009); and BP (Olsen et al. 2004).

Results from this study render NFAP2 of antifungal highly interesting compounds for development of a new antifungal strategy against yeasts after further studies focusing on its bulk production, antifungal mechanism, toxicity and in vivo activity.

## Additional file


10.1186/s13568-016-0250-8 Supplementary materials. **Fig. S1** Purity of the NFAP2 after the ion-exchange chromatography, checked with 18 % (w/v) tris–glycine sodium dodecyl sulfate–polyacrylamide gel (Novex™ 18 % Tris–Glycine Mini Protein Gels, 1.0 mm, 10-well; Thermo Fisher Scientific, Waltham, MA, USA) electrophoresis. **Table S1** Putative NFAP2 homologs from annotated filamentous Acomycota genomes. **Fig. S2** Alignment of the putative NFAP2 homolog proteins from filamentous ascomycetes. **Fig. S3** Bootstrap values of the maximum likelihood tree presented in Fig. 3b.

## Abbreviations

AMB: amphotericin B
ANAFP: *Aspergillus niger* antifungal protein
BLAST: basic local alingment search tool
BP: *Penicillium brevicompactum* bubble protein
CSP: caspofungin
ECD: electronic circular dichroism
ESI-MS: electrospray ionization mass spectrometry
FITC: fluorescein isothiocyanate
FLC: fluconazole
FPAP: *Fusarium polyphialidicum* antifungal protein
GRAVY: grand average of hydropathy
ITC: itraconazole
LCM: low cationic broth medium
MEA: malt extract agar
MIC: minimal inhibitory concentration
ML: maximum likelihood
MM: minimal medium
MS: mass spectrometry
NAC: non-albicans *Candida*
NFAP: *Neosartorya fischeri* antifungal protein
NFAP2: *Neosartorya fischeri* antifungal protein 2
PAF: *Penicillium chrysogenum* antifungal protein
PI: propidium iodide
RP-HPLC: reversed-phase high performance liquid chromatography
TRB: terbinafine
YEGK: yeast extract glucose medium
