# Supplementary material for: NFAP2, a novel cysteine-rich anti-yeast protein from Neosartorya fischeri NRRL 181: isolation and characterization
Source: AMB Express. 2016 Sep 15;6:75. doi: 10.1186/s13568-016-0250-8 (PMC5025423; doi:10.1186/s13568-016-0250-8)
Supplement: Supplementary file 1 — 10.1186/s13568-016-0250-8 Supplementary materials. Fig. S1 Purity of the NFAP2 after the ion-exchange chromatography, checked with 18 % (w/v) tris–glycine sodium dodecyl sulfate–polyacrylamide gel (Novex™ 18 % Tris–Glycine Mini Protein Gels, 1.0 mm, 10-well; Thermo Fisher Scientific, Waltham, MA, USA) electrophoresis. Table S1 Putative NFAP2 homologs from annotated filamentous Acomycota genomes. Fig. S2 Alignment of the putative NFAP2 homolog proteins from filamentous ascomycetes. Fig. S3 Bootstrap values of the maximum likelihood tree presented in Fig. 3b. [file 13568_2016_250_MOESM1_ESM.pdf]

Supplementary materials

AMB Express

NFAP2, a novel cysteine-rich anti-yeast protein from *Neosartorya fischeri* NRRL 181: Isolation and characterization

Liliána Tóth[1], Zoltán Kele[2], Attila Borics[3], László G. Nagy[3], Györgyi Váradi[2], Máté Virágh[1], Miklós Takó[1], Csaba Vágvölgyi[1], László Galgóczy[1,4*]

[1]Department of Microbiology, Faculty of Science and Informatics, University of Szeged, Közép fasor 52, Szeged H-6726, Hungary

[2]Department of Medical Chemistry, Faculty of Medicine, University of Szeged, Dóm tér 8, Szeged H-6720, Hungary

[3]Institute of Biochemistry, Biological Research Centre, Hungarian Academy of Sciences, Temesvári krt 62, Szeged H-6726, Hungary

[4]Division of Molecular Biology, Biocenter, Innsbruck Medical University, Innrain 80-82, Innsbruck A-6020, Austria

*Correspondence: galgoczi@gmail.com

[1]Department of Microbiology, Faculty of Science and Informatics, University of Szeged, Közép fasor 52, Szeged H-6726, Hungary

[4]Division of Molecular Biology, Biocenter, Innsbruck Medical University, Innrain 80-82, Innsbruck A-6020, Austria

Table S1 Putative NFAP2 homologs from annotated filamentous *Ascomycota* genomes

Fungus	Name of the protein	Acc. number or protein ID*	Sequence	Additional information	Database
Acremonium chrysogenum ATCC 11550	hypothetical protein ACRE_009950	KFH48093.1	IATDAYYACNCPNNCGHKEGSSCKFYSGPSDNSGIISGHCHYPNGNPMGAKECVP	n.d.	NCBI
Acremonium strictum DS1bioAY4a	gm1.11729_g	1373459	IATDAYYACNCPNNCGHSEGSSCKYYGGPSDTDDIISGTCNYPNGNRFASIECVA	n.d.	JGI
	gm1.6463_g	1368193	IATDPFFACQCPNNCDHSSGSSCKFYGGPSDSSNVVDGHCTDTADGLKCI		
Alternaria brassicicola	AB02612.1	2612	IAPDAINACNCPNNCQHKLGDSCKFYKEGYVVTAICRATDDDGVLECR	n.d.	JGI
Aspergillus fumigatus var. RP-2014	hypothetical protein BA78_8705	KEY83917.1	IATSPYYACNCPNNCKHKKGSSCKYHSGPSDKSKVISGKCEWQGGQLNCIAT	Origin: Brazil.<br>Isolation source: tropical forest floor.<br>Collection date: 2007.	NCBI
Aspergillus fumigatus Z5	hypothetical protein Y699_05481	KMK56566.1	IATSPYYACNCPNNCKHKKGSSCKYHSGPSDKSKVISGKCEWQGGQLNCIAT	Origin: China, Nanjing.<br>Isolation source: compost heaps.<br>Collection date: Mar 2010.	NCBI
Byssothecium circinans CBS 675.92	fgenesh1_kg.8_#_209_#_Locus10000v1rpkm3.04	489823	IAPDPIAACNCPNNCRHKNGSSCKFYDNGNTISGECQIEGDHLKCVA	Origin: USA, South Dakota.<br>Isolation source: rotten crown.<br>Substrate (including host): *Medicago sativa*.	JGI
Claviceps purpurea strain 20.1	uncharacterized protein CPUR_03466	CCE29619.1	IATDPFFACNCPNNCKHNEGTSCRYYSNYSDSGPVSRGKCGWKNGQLYCYA	n.d.	NCBI
	uncharacterized protein CPUR_03467	CCE29620.1	IATDAYHACNCPNNCGHKSGTGCRFFAGPSSNSNVLTGKCESVGGYLSCIAGPAQ		
	uncharacterized protein CPUR_03481	CCE29634.1	IATDAYHACNCPNNCSYRSGSSCRFRGGPSGNAPVLKGKCEFVGGYLSCIAQ		
	uncharacterized protein CPUR_03482	CCE29635.1	IATDPYFACNCPNNCSHKEGSSCRYYKNFSDSGPVAKGKCGWQNGHLNCYA		
Coniochaeta ligniaria NRRL 30616	fgenesh1_kg.1_#_380_#_Locus11190v1rpkm0.72	626667	IATDPVSACNCPNNCSHKSGSSCKYYSGPSDSSPIDSGKCAWRGSTLICVV	n.d.	JGI
Coniochaeta sp. PMI_546	gm1.7183_g	970051	IATDPVSACNCPNNCSHKKGSSCRYYSGPSDSSPVDSGHCEWRGSTLICIV	Isolation source: *Populus deltoides*.	JGI
Daldinia eschscholzii EC12	gm1.7079_g	24471	IATNSVAACNCPNNCSHKENSSCKFYSGPSDTSKVVSGRCHSQNGVLTCIP	n.d.	JGI
Eutypa lata UCR-EL1	hypothetical protein UCREL1_925	EMR72015.1	IATDPGYACTCPNNCDHHAGSSCKYYSGPSDNSPIIEGTCVDRNGLTCVA	n.d.	NCBI
Hypoxylon sp. CI-4A	gm1.10063_g	16636	IATDAFSACNCPNNCDHSAGSSCKYHAGPSDSSKTVSGKCNKPNGNPYASLECIVTS	n.d.	JGI
Hypoxylon sp. CO27-5	gm1.426_g	26620	IAVDAVAACNCPNNCSHGNGSSCKYLAGPSTSSSVISGKCTPEADGTLICIPK	n.d.	JGI
	gm1.7264_g	33458	IATTAFAACNCPNNCDHKAGSDCKYYSGPSSKSDVLKGKCAKPNGNPYASIECIPN		
Hypoxylon sp. EC38	gm1.528_g	528	IAVDAVAACNCPNNCSHGNGSSCKYLAGPSTSSSVISGKCTPEADGTLICIPK	n.d.	JGI
	gm1.11636_g	11636	IATTAFAACNCPNNCEHKAGSDCKFYSGPSSKSDVLKGKCAKPNGNPYASIECIPN		
Karstenula rhodostoma CBS 690.94	gm1.14118_g	492330	IAPDPDAACNCPNNCQHSNGESCSFYRDGNHLDGICQHTGEGGRLLCVA	Origin: Sweden, Uppland, Dalby Parish, NW of farm Viggeby.<br>Substrate (including host): *Frangula alnus*.<br>Collection date: 25/02/1986.	JGI

Massarina eburnea CBS 473.64	gm1.2277_g	514471	IAADPVAACNCPGNCKHKNGDSCKFYENGNTLSGQCVTDGDHLKCVA	Country: Switzerland, Zürich, Talstrasse, Rehalp Forest.<br>Substrate (including host): *Fagus sylvatica*.	JGI
Melanomma pulvis-pyrius	fgenesh1_kg.370_#_2_#_Locus74v1rpkm1368.98	327945	IAPDPISACNCPNNCQHKVGSSCKFYSQGNTLSGECQLNGNAGNLICIA	n.d.	JGI
Myriangium duriaei CBS 260.36	gm1.4858_g	293795	IAPDPTSACNCPKNCSHHAGSSCKFYRNGNVITGVCNDVGGHLICQD	Origin: Argentina, Delta del Paraná.<br>Substrate (including host): *Chrysomphalus aonidium*.	JGI
Niesslia exilis CBS 358.70	estExt_Genemark1.C_1_t10048	854608	IATNPFYACNCPNNCDHGPGDSCKYYDGPSNTSPVVDGTCQSSGSATGWNCVPN	Origi: Netherlands, Noord Brabant, Kampina Heide.<br>Substrate (including host): *Pteridium aquilinum* petiole.	JGI
	estExt_Genemark1.C_190067	864148	IATTSYAACNCPNNCSYTQGSSCKYFAGPSDTDTIISGTCQYPNGNQAAEITCIA		
	gm1.8228_g	828052	IATDAYSACNCPNNCSYSQGTGCRYYSTPSNSGPVASGTCDYPGGNHNSGLTCIPSS		
Paraconiothyrium sporulosum AP3s5-JAC2a	estExt_Genemark1.C_5_t20273	1259469	IAANPDAACNCPNNCQHNNGDSCSFYRDGNQLNGICQHTGEGGRLLCNA	n.d.	JGI
Pseudogymnoascus pannorum VKM F-3808	hypothetical protein O988_03546	KFY00055.1	IATDPYYACNCPNNCSYKPGTGCRFYSGPSDNSKVLKGKCQNKNGKLTCIPT	Origin: Russia, Tverskaya oblast.<br>Isolation source: hair.	NCBI
Pseudogymnoascus pannorum VKM F-4513	hypothetical protein V494_01379	KFY44674.1	VATDPYYACNCPNNCSYKEGSGCRFYSGPSDNSRILKGKCHRKDGKLTCVP	Origin: Russia, Kolyma Lowland.<br>Isolation source: permafrost soil.	NCBI
Pseudogymnoascus pannorum VKM F-4514	hypothetical protein V495_02588	KFY46251.1	IATDPYYACNCPNNCSYKPGTGCRFYSGPSDNSKVLKGKCQNKNGKLTCIPT		
Pseudogymnoascus pannorum VKM F-4516	hypothetical protein V497_02880	KFY61541.1	IATDPYYACNCPNNCSYKPGTGCRFYSGPSDNSKVLKGKCQNKNGKLTCIPT		
Pyrenophora tritici-repentis	PTRT_08027	153706	IAPDATNACNCPNNCRHRVGDSCKFYQQGYVVTATCQQGGANGALICK	n.d.	JGI
Sordaria macrospora K-hell	hypothetical protein SMAC_09189	XP_003344816.1	IATDPFYACNCPNNCSHKAGSGCKYHSGPSDKSSVVKGKCVVRGGGLTCVSG	Tissue type: mycelium.	NCBI
Thozetella sp. PMI_491	gm1.14407_g	828875	IAPDPVAACNCPNNCKHHLGSSCKFYSNGNVLSGVCQHDGSGLKCAV	Origin: Australia.<br>Isolation source: bark.	JGI

Abbreviation of databases: NCBI, National Center for Biotechnology Information; Bethesda, MD, USA; JGI, Joint Genome Institute; Walnut Creek, CA, USA. *: In the cases of NCBI and JGI database the accession number and the protein ID is given, respectively

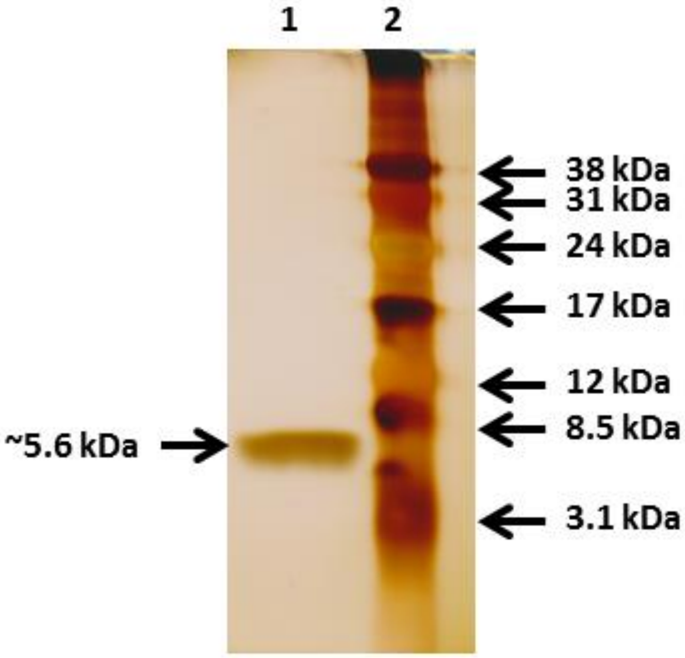

Fig. S1 Purity of the NFAP2 after the ion-exchange chromatography, checked with 18% (w/v) tris-glycine sodium dodecyl sulfate-polyacrylamide gel (Novex™ 18% Tris-Glycine Mini Protein Gels, 1.0 mm, 10-well; Thermo Fisher Scientific, Waltham, MA, USA) electrophoresis. Protein bands were detected with silver staining. Lane 1: purified NFAP2 (1.5 μg), lane 2: Low-range Amersham Rainbow Marker (GE Healthcare Life Sciences, Little Chalfont, UK)

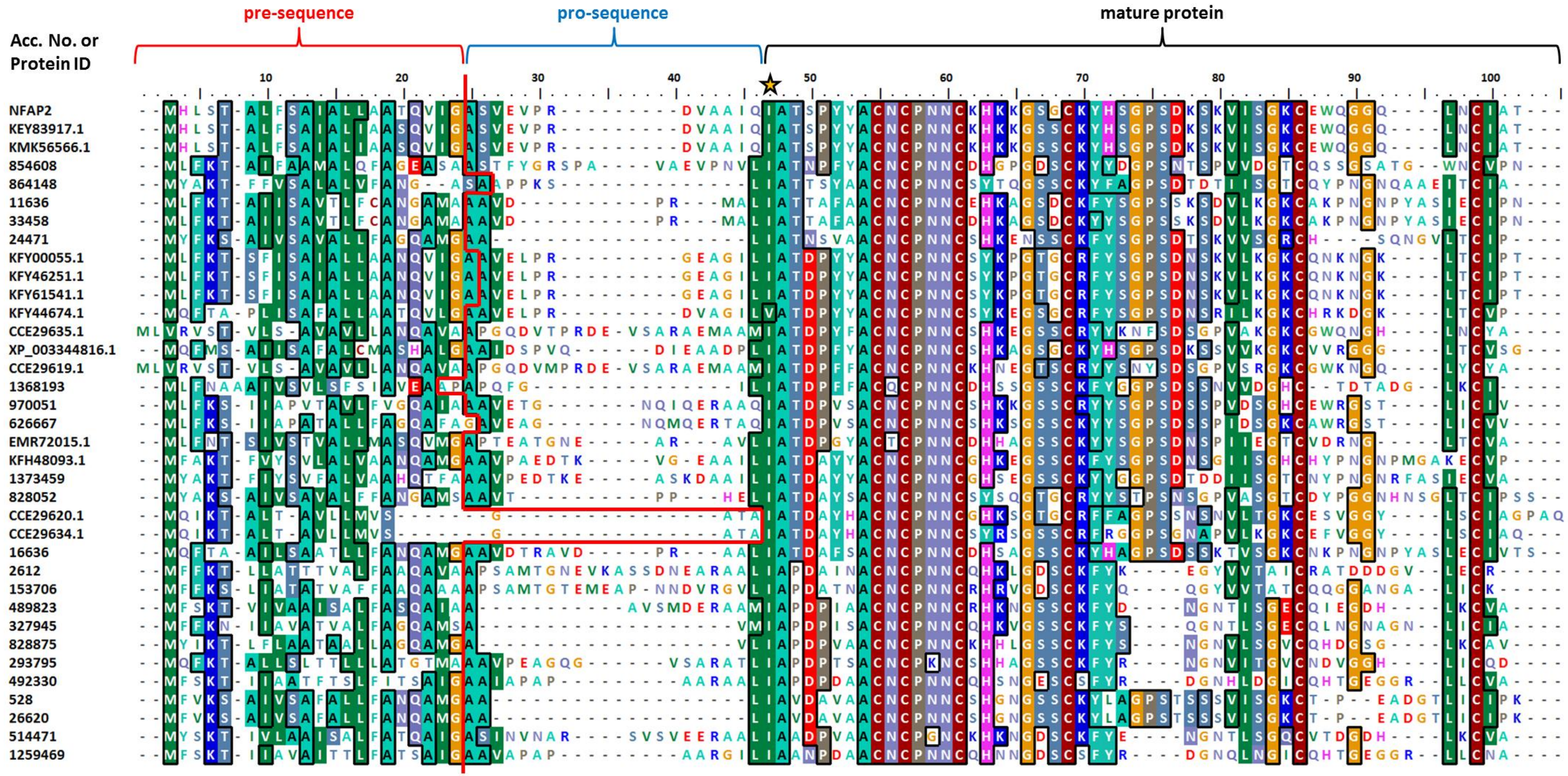

Fig. S2 Alignment of the putative NFAP2 homolog proteins from filamentous ascomycetes. Red line indicates the cleavage site of the predicted signal sequence (SignalP1 4.1 server; Petersen et al. 2011). First amino acid of the mature protein is marked by an asterisk. Accession number or the protein ID is indicated in the figure

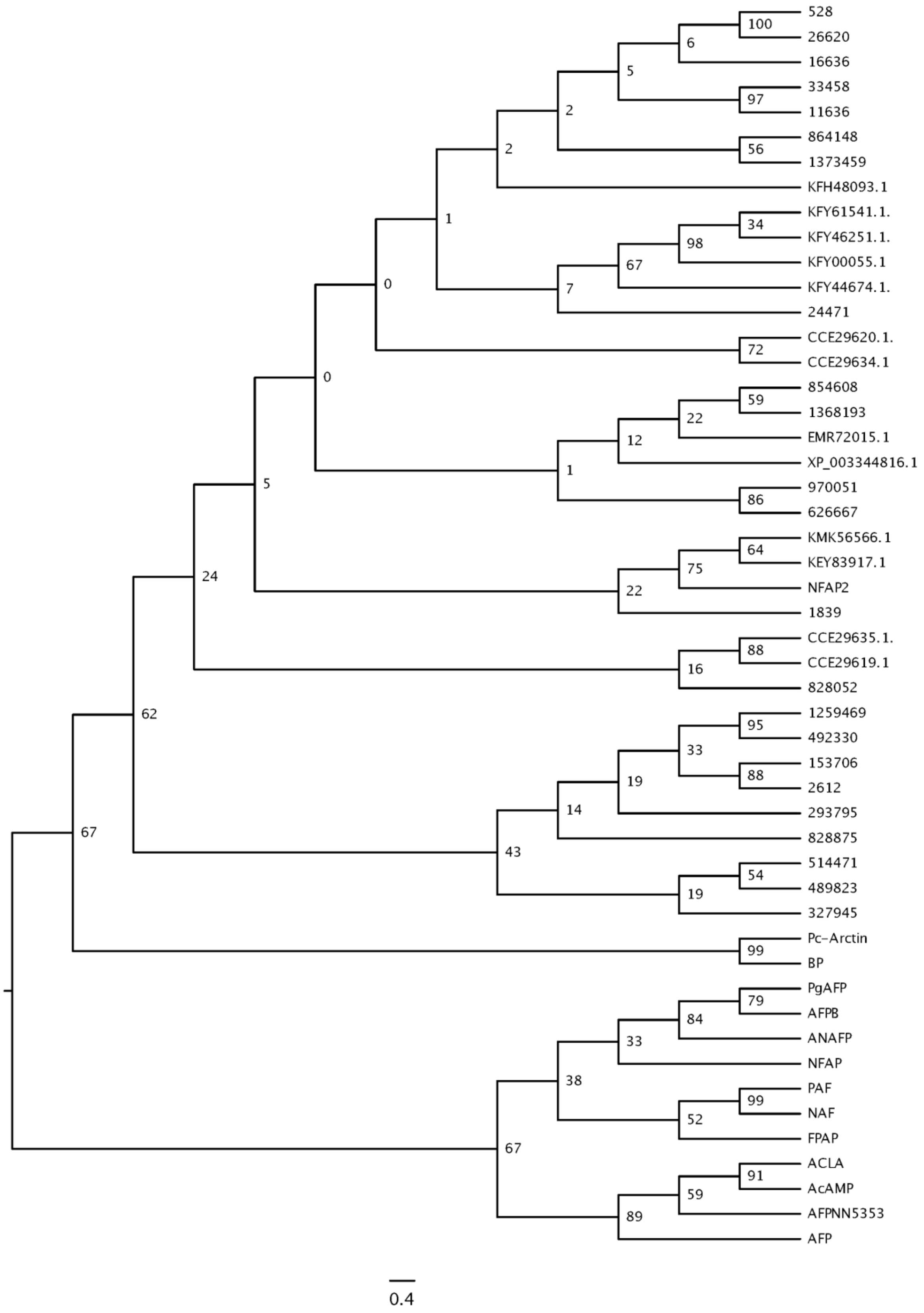

Fig. S3 Bootstrap values of the maximum likelihood tree presented in Fig. 3b

AcAFP, *Aspergillus clavatus* VR1 antifungal protein (Acc. No.: A1CSS4); AcAMP, *Aspergillus clavatus* ES1 antimicrobial peptide (Acc. No.: D3Y2M3); AFP, *Aspergillus giganteus* MDH 18894 antifungal protein (Acc. No.: P17737); AFP_{NN5353}, *Aspergillus giganteus* A3274 antifungal protein (Acc. No.: -); AFPB, *Penicillium digitatum* CECT 20796 antifungal protein (Acc. No.: K9FGI7); ANAFP, *Aspergillus niger* KCTC 2025 antifungal protein (Acc. No.: A2QM98); BP, *Penicillium brevicompactum* Dierckx 'bubble protein' (Acc. No.: G5DC88); FPAP, *Fusarium polyphialidicum* SZMC 11042 antifungal protein (Acc. No.: E1UGX4); NAF, *Penicillium nalgiovense* BFE 66, 67, 474 antifungal protein (Acc. No.: -); NFAP, *Neosartorya fischeri* NRRL 181 antifungal protein (Acc. No.: D4YWE1); NFAP2, *Neosartorya fischeri* NRRL 181 antifungal protein 2 (Acc. No.: A1DBL3); PAF, *Penicillium chrysogenum* Q176 antifungal protein (Acc. No.: B6HWK0); Pc-Arctin, *Penicillium chrysogenum* A096 'bubble protein' (Acc. No.: CAP96194); PgAFP, *Penicillium chrysogenum* RP42C antifungal protein (Acc. No.: D0EXD3). The Acc. No. or Protein ID of the putative NFAP2 homolog proteins is indicated. For further information (species name, sequence, etc.) see Table S1
